# Practice Nurse Provision of Long‐Acting Reversible Contraception: A Cross‐Sectional Survey of Knowledge and Practices

**DOI:** 10.1111/jan.17020

**Published:** 2025-05-01

**Authors:** Sharon James, Satish Melwani, Stella May Gwini, Kirsten I. Black, Angela Taft, Deborah Bateson, Wendy V. Norman, Danielle Mazza

**Affiliations:** ^1^ Department of General Practice, School of Public Health and Preventive Medicine, SPHERE, NHMRC Centre of Research Excellence Monash University Melbourne Australia; ^2^ Monash Centre for Occupational & Environmental Health, School of Public Health and Preventive Medicine Monash University Melbourne Australia; ^3^ Specialty of Obstetrics, Gynaecology and Neonatology, Faculty of Medicine and Health University of Sydney Sydney Australia; ^4^ School of Nursing and Midwifery, Judith Lumley Centre LaTrobe University Melbourne Australia; ^5^ Faculty of Medicine and Health University of Sydney Sydney Australia; ^6^ Department of Family Practice University of British Columbia Vancouver Canada

**Keywords:** contraception, long‐acting reversible contraception, nursing, primary care, women's health

## Abstract

**Aim:**

To describe practice nurse long‐acting reversible contraception (LARC) knowledge and practices.

**Design:**

Cross‐sectional survey.

**Methods:**

Between July and December 2021, we conducted an online survey using convenience sampling to recruit Australian nurses who work in primary care, known as practice nurses. We collected data about demographics and knowledge and practices relating to LARC. Analysis used descriptive statistics and Poisson regression.

**Results:**

From 489 eligible responses, most respondents were women and the majority worked in metropolitan practices. Most (90.4%) believed that their advice could influence women's contraceptive choices. Few inserted/removed intrauterine devices (IUDs) (11.2%) or implants (15.9%). Of those that did insert LARC, most did so one to five times in the last month (IUDs 72.2%; implants 73.6%). General practice as a primary place of work was negatively associated with implant provision. Respondents with more general practice experience (≥ 15 years) and/or higher qualifications were more likely to respond correctly to knowledge questions and provide IUDs or implants. Most (62.8%) correctly identified IUD suitability for nulliparous women.

**Conclusions:**

Practice nurses have knowledge gaps and limited practice opportunities for LARC provision.

**Implications:**

Practice nurses need supportive funding policies and ongoing education and skills development to enhance patient access to LARC and their choice of provider.

**Reporting Method:**

CHERRIES guideline.

**Patient or Public Contribution:**

Partner organisations assisted with the study's recruitment.

**Trial Registration:**

ACTRN12622000655741

**Summary**

- Our study examined practice nurse knowledge and practices about LARC.
- Practice nurses experience knowledge gaps and limited opportunities for providing LARC.
- Policymakers have the opportunity to better support and utilise practice nurses to enhance patient access to LARC.
- The paper delivers the evidence base about the need to better utilise practice nurses to provide LARC.

## 1 Introduction

Maximising the practice scope of nurses providing contraception makes it more likely that these services are available to the women^1^ who want it (Mazza et al. 2023). This allows population groups, particularly those more at risk of unintended pregnancy (e.g., those living in rural areas, culturally and linguistically diverse populations and/or adolescents), to be better able to access this care (Bittleston et al. 2024; Rowe et al. 2016). Ensuring nurses are appropriately trained, mentored and enabled to provide effective contraception will support women's decision‐making about their sexual and reproductive healthcare needs. The most effective contraceptive methods, long‐acting reversible contraception (LARC), require minimal user adherence (The Faculty of Sexual and Reproductive Healthcare 2019). LARC methods, intrauterine devices (IUDs) and contraceptive implants can be in place for 3–10 years depending on the method used (The Faculty of Sexual and Reproductive Healthcare 2019).

Most women spend over three quarters of their reproductive years trying to prevent a pregnancy (Guttmacher Institute 2019). Choice about whether to become pregnant, the number of children and spacing between pregnancies is a human right (United Nations Department of Economic and Social Affairs Population Division 2022). Reproductive autonomy is identified through the Sustainable Development Goals to ensure universal access to sexual and reproductive health (SRH) services, such as family planning and the incorporation of reproductive health into national policies and programmes (United Nations Department of Economic and Social Affairs Population Division 2022). Even so, there are approximately 164 million women trying to prevent or postpone pregnancy who have unmet contraception needs (United Nations Department of Economic and Social Affairs Population Division 2022).

Equitable and timely access to contraception empowers women, including adolescents, to pursue educational and employment opportunities and supports country‐based benefits including sustainable population growth and economic development (World Health Organization 2020). Contraceptive use consists of short‐acting methods (45%) (e.g., male condoms and oral contraceptives), sterilisation (25%), LARC (i.e., contraceptive implant and IUDs) (19%) and traditional/other methods (11%) (e.g., withdrawal and rhythm method) (United Nations Department of Economic and Social Affairs Population Division 2022). The health and social benefits of access to effective contraceptive methods include the management of interpregnancy intervals, unintended pregnancy and heavy menstrual bleeding (World Health Organization 2020).

## 2 Background

Preventing unintended pregnancy is a key focus of Australia's National Women's Health Strategy 2020–2030 (Australian Government Department of Health 2018a). Almost one‐third (30.4%) of all unintended pregnancies end in abortion, with women in rural and regional Australia 1.4 times more likely to experience an unintended pregnancy than those living in metropolitan settings (Rowe et al. 2016; Taft et al. 2018). The main reasons for unintended pregnancies include contraceptive failure and absent or inconsistent contraceptive use (Rowe et al. 2016).

Contraceptive use in Australia is skewed towards less effective forms of contraception, such as oral contraceptives (37.2%) and condoms (33.9%) (Richters 2016). Uptake of LARC, whilst 99% effective, is low in Australia (10.8%) compared to some European nations (up to 32%) (Eeckhaut et al. 2014; Grzeskowiak et al. 2021). Access to LARC can be problematic for those residing in non‐urban settings due to lack of health professionals providing these services (Mazza et al. 2017). Women experience increased travel time and associated costs of LARC insertion/removal, community and clinician misconceptions about LARC contraceptive methods and concerns about contraceptive coercion (Linton et al. 2023; Mazza et al. 2017).

In Australia, LARC services are largely provided in primary healthcare (PHC) settings by general practitioners (GPs) through general practices (also known as primary care or family practice), sexual health or family planning services (Royal Australian and New Zealand College of Obstetricians and Gynaecologists 2017). General practice is the most accessed healthcare setting (The Royal Australian College of General Practitioners 2024). While all nurses (i.e., enrolled and registered nurses) and midwives can provide LARC care, additional training is required to insert/remove LARC that is over and above their initial qualification. In Australia, LARC insertion/removal in the literature largely focuses on registered nurses, nurse practitioners and midwives (Botfield et al. 2019; James et al. 2023). To support patient choice for health professionals involved in SRH care, contraceptive options and heavy menstrual bleeding management, strengthening nursing training and roles in LARC care is in line with national workforce and public health initiatives (Australian Government Department of Health 2018b).

There are approximately 14,360 practice nurses (PNs) in Australia (Australian Government Department of Health and Aged Care 2022). PNs work in 92% of general practice settings (The Royal Australian College of General Practitioners 2023), and have the potential to further enable women's access to LARC services. These PNs are a regulated workforce with diploma qualifications as an enrolled nurse, baccalaureate (or equivalent) registered nurse or as a Masters qualified and clinically experienced nurse practitioner (Australian Government Department of Health and Aged Care 2023). Internationally and in Australia, the PN role broadly includes triage, prevention and health promotion, care coordination, treatment, acute and ongoing care (Australian Primary Health Care Nurses Association, n.d.; Health Education England 2017; Mid‐Central District Health Board and New Zealand Nurses Organisation Tōpūtanga Tapuhi Kaitaki O Aotearoa and the New Zealand College of Primary Health Care Nurses 2014 (Updated 2019)). In Australia, this care is largely undertaken on behalf of the GP, supported by government funded clinical encounters and block funding and/or care paid for by the patient (Freund et al. 2015). Most care provided by PNs has historically aligned with these funding mechanisms; predominantly for the management of chronic conditions (Freund et al. 2015).

Existing SRH literature identifies PHC nursing roles in sexual health promotion, medication abortion, cervical screening, as well as sexually transmitted infection testing and follow‐up (Horwood et al. 2020; Mazza et al. 2023; Mills et al. 2012; Wood et al. 2021). Although there is opportunity to enhance women's access to the most effective methods of contraception, LARC, there is limited local and international literature about SRH care provided by PNs working in general practice (Hoggart et al. 2018; Mazza et al. 2023).

## 3 The Study

### 3.1 Aim and Research Question

We aimed to describe Australian PN LARC knowledge and practices and associations between their characteristics and LARC knowledge and practice to answer the research question ‘What are Australian PNs' knowledge and practices about LARC?’.

## 4 Methods

### 4.1 Design

This survey was conducted as a part of the Australian Contraception and Abortion Primary Care Practitioner Support (AusCAPPS) Network Trial (ACTRN12622000655741) (Mazza et al. 2022). The AusCAPPS Network is a multidisciplinary national online community of practice, established to improve patient access to LARC and medication abortion by supporting Australian primary care clinicians providing these services (Mazza et al. 2022). Our survey was launched at the same time as the AusCAPPS Network as a national online cross‐sectional survey involving primary care clinicians (PNs, GPs and community pharmacists). Our aim was to determine these clinicians' baseline measures of medication abortion and LARC knowledge, attitudes and practices. Given the volume of data, this paper only examines PNs LARC knowledge and practices. The Checklist for Reporting Results of Internet E‐Surveys (CHERRIES) was used to direct the reporting of this survey (Eysenbach 2004) (File S1).

### 4.2 Study Setting and Sample

We aimed to recruit a sample of 500 PNs working in Australian general practice (where the estimated population was 14,360 PNs (Australian Government Department of Health and Aged Care 2022)) to estimate the proportion of participants with key characteristics and LARC knowledge and practices with a precision of ±5% and assuming 50% of PNs were knowledgeable of LARC. Survey recruitment methods included e‐mails to research team contacts and professional organisations including the Australian PHC Nurses Association, and mail outs using a purchased health professional address list. Recruitment was also conducted on social media platforms including LinkedIn, Twitter (now X) and Facebook sites such as the Australian General Practice Nurses Network.

### 4.3 Inclusion and/or Exclusion Criteria

All respondents were registered (licenced) and verified against the Australian Health Practitioner Regulation Agency (AHPRA) website.

### 4.4 Instrument

Based on prior surveys about LARC and medication abortion provision (Jones et al. 2017; Mazza et al. 2016; Norman et al. 2016), this ‘open’ online survey was developed and piloted by clinicians and academics with experience in primary care and women's health. Taking approximately 10–15 min to complete, respondents could answer a maximum of 50 questions over nine pages. Respondents could revisit their responses prior to submission. The survey was written in English.

Demographic questions included age, gender, practice postcode, and years practising as a PN. Questions relating to contraception knowledge and provision were compulsory. These included questions on whether or not the nurses inserted or removed LARC or provided counselling on LARC. If nurses did provide LARC they were asked how often/how many per month. They were also asked additional questions designed to test their knowledge and attitudes towards LARC. The LARC questions were chosen based on literature and anecdotal common misperceptions in knowledge about LARC (i.e., IUDs are unsuitable in nulliparous women and the slow return of fertility post LARC removal) and LARC practice (i.e., insertion/removal). More details on the questions asked can be found in File S2. Free‐text fields were offered where respondents had selected ‘other’ responses, such as ‘Please describe where you have received your IUD training’. Respondents were invited to join the AusCAPPS Network following survey completion.

### 4.5 Data Collection and Data Analysis

Survey completion was voluntary. Data were collected between July and December 2021. All survey responses were captured and stored in REDCap (Research Electronic Data Capture), hosted and managed by Helix (Monash University) (Harris et al. 2019, 2009). Survey data verification and cleaning was conducted in Microsoft Excel. We offered a $AUD40 e‐gift card for survey completion. While cookies, IP and geolocation REDCap features were not enabled, the research team conducted a verification process to screen respondents (including matching the survey respondent's name to their registration status on the AHPRA database) and included fraud mitigation strategies for example, a combination of human and computer‐based approaches. This included a reCAPTCHA, honeypot question only recognisable by computer and screening by the research team for illogical, duplicate or fast responses (i.e., < 5 min for completion).

Rurality was determined using the Modified Monash Model (Australian Government Department of Health and Aged Care 2021), calculated from the respondent's postcode. Data were analysed using STATA 18 (StataCorp 2023). Descriptive characteristics of nurses, their knowledge and practices were summarised as frequencies with percentages. We used Poisson regression, with robust error sandwich estimators, to test for the association between LARC knowledge and practices (as dependent variables) with demographic factors (e.g., age, rurality and primary place of work) as independent variables. Only knowledge/practice questions with sufficient variability (i.e., with no more than 80% of PNs with one response) were assessed. Results were then reported as risk ratios together with the 95% confidence interval. A *p* value of < 0.05 was considered statistically significant. Given the limited description required for open text responses, these were grouped by key ideas and presented in tables within ‘Other’ responses.

### 4.6 Ethical Considerations

The Monash University Human Research Ethics Committee (No. 28002) approved the study. Prior to survey commencement, an explanatory statement was available for prospective respondents. Survey completion indicated implied consent. As per Monash University policy, all confidential data were only accessible by the research team and were stored on a password‐protected file within the University's secured internal network.

## 5 Results

### 5.1 Description of Study Sample

**FIGURE 1:**
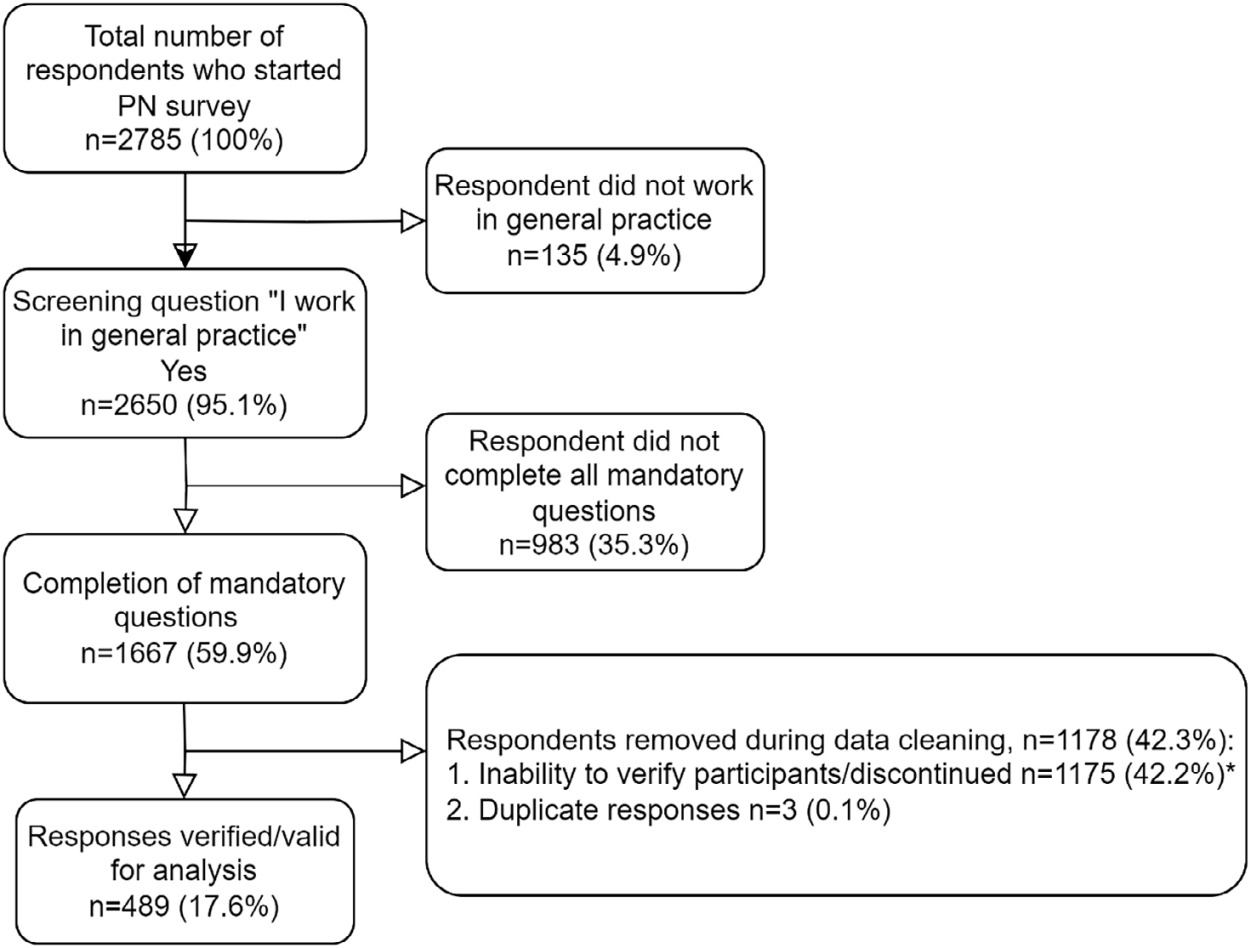
Response flow diagram. *Differentiation between discontinued surveys from some fraudulent responses may not be possible as respondents may have discontinued the survey at differing stages. In addition, the honeypot question was implemented as part of the verification process after survey launch and positioned prior to the respondent indicating their professional group.

This study was part of a larger survey with other health professional groups. For the purposes of this analysis, respondents who selected PN were considered unique survey visitors. Among 2785 responses, 489 responses were valid. Based on workforce data of 14,360 PNs and the total number of valid responses, this study sample represents approximately 3.4% of the PN workforce (Australian Government Department of Health and Aged Care 2022). Excluded responses included those who did not work in general practice (*n* = 135), incomplete surveys (*n* = 983), an inability to verify respondent AHPRA status (*n* = 1175) or where duplicate responses were recorded (*n* = 3). Calculated from all users who completed the first survey question and all users who completed the final mandatory question (*n* = 1828), the completion rate was 68.9%. Figure 1 identifies rates of participation at each stage of the response flow.

### 5.2 Respondent Demographic Characteristics

From 489 valid responses, most reported being women (96.7%) and most worked in metropolitan practices (63.3%) (Table 1). Respondents were mostly aged 50–59 years (26.4%). Most had worked in primary care for 0–4 years (34.4%). Their primary place of work was largely general practice (93.1%) followed by ‘other’ (4.1%) and those that indicated a secondary place of work mainly indicated ‘other’ for example, hospital, community health or another general practice. Respondents were predominantly qualified as registered nurses (70.1%). One in 10 respondents indicated they could consult in a language other than English (10.6%; *n* = 52).

**TABLE 1:** Demographic characteristics of practice nurses (*N* = 489).

	% (*n*)
**Age**	
18–24	4.7 (23)
25–29	10.4 (51)
30–34	11.3 (55)
35–39	12.7 (62)
40–44	10.6 (52)
45–49	10.8 (53)
50–54	14.1 (69)
55–59	12.3 (60)
60–64	10.2 (50)
65+	2.9 (14)
**Gender**	
Woman	97.6 (477)
Man	1.8 (9)
Non‐binary	0.4 (2)
Prefer not to answer	0.2 (1)
**Rurality**	
1 (Metropolitan)	63.3 (307)
2–5 (Regional/Rural)	34.9 (169)
6–7 (Remote/Very Remote)	1.9 (9)
**Primary place of work**	
General practice	93.1 (455)
Other for example, aboriginal, defence or university health services	4.1 (20)
Women's health service	1.6 (8)
Family planning organisation	0.6 (3)
Refugee health	0.6 (3)
**Years worked in general practice**	
0–4	34.4 (168)
5–9	27.8 (136)
10–14	18.8 (92)
15–19	8.6 (42)
20–24	5.3 (26)
25–29	2.9 (14)
30+	2.3 (11)
**General practice type**	
Small business	66.9 (327)
Corporate chain	16.0 (78)
Other eg. Aboriginal Health Corporation, Community Health Service	9.8 (48)
Not stated	7.4 (36)
**Qualifications held** *(multiple choices allowed)*	
Enrolled nurse	9.4 (46)
Registered nurse	70.1 (343)
Registered nurse (advanced practice)	14.5 (71)
Nurse practitioner	7.2 (35)
Other for example, midwife, child and family health nurse	3.9 (19)
**Consults in language other than English**	
No	89.4 (437)
Yes	10.6 (52)

### 5.3 Knowledge About LARC Methods

Most (86.5%) respondents recognised LARC's effectiveness compared to oral contraceptive options (Table 2). Just under two‐thirds (62.8%) correctly identified that IUDs are suitable for use in nulliparous women, and just under a third (28.8%) were unsure of their suitability.

**TABLE 2:** Knowledge about LARC methods.

	% (*n*)
*Knowledge of LARC*	
Long‐acting reversible contraceptives are less effective than the contraceptive pill at preventing pregnancy—False	
True	4.5 (22)
False	86.5 (423)
Unsure	9.0 (44)
Intrauterine devices' are suitable for use in nulliparous women—True	
True	62.8 (307)
False	8.4 (41)
Unsure	28.8 (141)
Nurse/nurse practitioners' views and advice can influence the type of contraception selected by patients—True	
True	90.4 (442)
False	3.7 (18)
Unsure	5.9 (29)
Fertility can return rapidly after long‐acting reversible contraceptive removal—True	
True	76.7 (375)
False	10.2 (50)
Unsure	13.1 (64)
Factors that influence LARC recommendation	
Age	66.1 (323)
Cost	64.6 (316)
Patient's BMI	24.7 (121)
Past history of abnormal cytology	21.1 (103)
Past history of sexually transmitted infections	20.3 (99)
Marital status	14.5 (71)
History of full‐term pregnancies	18.4 (90)
History of abortion	37.8 (185)
Clinician preference	19.2 (94)
I don't recommend LARC	17.4 (85)
Other	11.0 (54)

Respondents aged > 60 years, those with ≥ 10 years of experience in general practice and those qualified as registered nurses/(advanced practice) registered nurses/nurse practitioners were more likely to be aware of the suitability of IUDs for nulliparous women when compared to those aged < 30 years, those with < 5 years of experience in general practice and/or those qualified as an enrolled nurse, respectively (Table 3).

**TABLE 3:** Relationship between respondent characteristics and LARC knowledge and practices.

Characteristic	Knowledge				Practices			
	IUDs' suitability in nulliparous women (true)		Fertility returns post LARC removal (true)		Insert/remove IUDs		Insert/remove implants	
	Row % (*n*)	RRR (95% CI)	Row *n* (%)	RRR (95% CI)	Row *n* (%)	RRR (95% CI)	Row *n* (%)	RRR (95% CI)
Age								
< 30	58.1 (43)	Ref	68.9 (51)	Ref	10.8 (8)	Ref	21.6 (16)	Ref
30–39	53.9 (63)	0.93 (0.72–1.20)	80.3 (94)	1.17 (0.98–1.39)	13.7 (16)	1.26 (0.57–2.81)	17.1 (20)	0.79 (0.44–1.43)
40–49	58.1 (61)	1.00 (0.78–1.29)	77.1 (81)	1.12 (0.93–1.35)	8.6 (9)	0.79 (0.32–1.96)	20.0 (21)	0.93 (0.52–1.65)
50–60	70.5 (91)	1.21 (0.97–1.52)	77.5 (100)	1.12 (0.94–1.35)	19.4 (25)	1.79 (0.85–3.77)	20.9 (27)	0.97 (0.56–1.68)
60+	76.6 (49)	**1.32 (1.04–1.67)**	76.6 (49)	1.11 (0.91–1.36)	12.5 (8)	1.16 (0.46–2.91)	15.6 (10)	0.72 (0.35–1.48)
Rurality								
Other	64.0 (114)	Ref	74.2 (132)	Ref	15.7 (28)	Ref	21.4 (38)	Ref
Metropolitan	61.6 (189)	0.96 (0.83–1.11)	77.6 (239)	1.05 (0.94–1.16)	12.1 (37)	0.77 (0.49–1.21)	17.9 (55)	0.84 (0.58–1.22)
Primary place practice								
Other	76.5 (26)	Ref	79.4 (27)	Ref	17.7 (6)	Ref	35.3 (12)	Ref
General practice	61.8 (281)	0.81 (0.66–0.99)	76.5 (348)	0.96 (0.81–1.15)	13.2 (60)	0.75 (0.35–1.60)	18.0 (82)	**0.51 (0.31–0.84)**
Years in general practice								
0–4	51.2 (86)	Ref	71.4 (120)	Ref	7.1 (12)	Ref	14.3 (24)	Ref
5–9	61.0 (83)	1.19 (0.98–1.45)	75.0 (102)	1.05 (0.92–1.20)	8.8 (12)	1.23 (0.57–2.66)	15.4 (21)	1.08 (0.63–1.86)
10–14	72.8 (67)	**1.42 (1.17–1.73)**	84.8 (78)	**1.19 (1.04–1.35)**	13.0 (12)	1.83 (0.85–3.90)	13.0 (12)	0.91 (0.48–1.74)
≥ 15	76.3 (71)	**1.49 (1.24–1.80)**	80.6 (75)	1.13 (0.98–1.30)	20.4 (19)	**2.86 (1.45–5.63)**	22.6 (21)	1.58 (0.93–2.68)
Highest qualifications held								
Enrolled nurse (*N* = 46)	34.8 (16)	Ref	65.2 (30)	Ref	8.7 (4)	0.81 (0.30–2.17)	15.2 (7)	1.11 (0.53–2.31)
Registered nurse (*N* = 340)	63.6 (213)	**1.83 (1.22–2.74)**	75.8 (254)	1.16 (0.93–1.45)	10.8 (36)	Ref	13.7 (46)	Ref
Registered nurse (advanced practice) (*N* = 76)	67.6 (48)	**1.94 (1.27–2.98)**	83.1 (59)	**1.27 (1.01–1.61)**	22.5 (16)	**2.10 (1.23–3.57)**	33.8 (24)	**2.46 (1.61–3.76)**
Nurse practitioner (*N* = 36)	80.0 (28)	**2.30 (1.50–3.53)**	91.4 (32)	**1.40 (1.11–1.77)**	28.6 (10)	**2.66 (1.45–4.89)**	48.6 (17)	**3.54 (2.29–5.46)**

*Note:* Bolded results indicate significant findings at *p*<0.05.

Most (76.7%) respondents correctly indicated that fertility can return rapidly after LARC removal (Table 2). Correct knowledge about fertility return was more likely among respondents with 10–14 years of experience in general practice and those with the highest qualifications ([advanced practice] registered nurses and nurse practitioners) when compared to those with < 5 years of experience in general practice and/or those qualified as an enrolled nurse, respectively (Table 3).

A high proportion of participants (90.4%) believed that their views and advice can influence the type of contraception selected by patients (Table 2). Factors that influence a nurse recommending LARC methods to an eligible patient were largely related to patient age (66.1%), cost (64.6%) and history of abortion (37.8%). For the possible side effects of LARC, over half of the respondents agreed that the benefits outweighed the possible side effects of IUDs (57.4%; *n* = 280) and implants (61.8%; *n* = 302).

### 5.4 LARC Practices

Few respondents provided insertion or removal of IUDs (11.2%) or implants (15.9%) (Table 4). Of those that did insert IUDs or implants, most did so on 1–5 occasions in the last month preceding survey completion. Respondents with ≥ 15 years of experience in general practice were more likely to insert/remove IUDs, compared with those < 5 years' experience (Table 3). Compared with enrolled and registered nurses, advanced practice registered nurses and nurse practitioners were more likely to insert/remove both IUDs and implants. However, those whose primary place of work was general practice were less likely to insert/remove implants (Table 3). Few respondents had undertaken LARC training (IUD: 6.5%; implant 18.2%). Respondents who had undertaken LARC training largely did so through a family planning organisation (IUD: 78.1%; implant 59.6%) (Table 4).

**TABLE 4:** LARC services provided by PNs.

	% (*n*)
Insert/remove IUDs	
I insert and remove	3.7 (18)
I only insert	0 (0)
I only remove	7.6 (37)
Neither	88.8 (434)
Number of IUDs inserted in the last month (*n* = 18)	
< 1	0.0 (0)
1–5	72.2 (13)
> 5	27.8 (5)
Received IUD training	
No	93.5 (457)
Yes	6.5 (32)
Type of IUD training received (*n* = 32)	
Family Planning course	78.1 (25)
Other (GP supervisor, SHINE, Mentors)	21.9 (7)
Where do women you consult with have IUD insertions?	
GP in my practice	65.2 (319)
Private gynaecologist	28.0 (137)
Public hospital	18.0 (88)
Family planning clinic	16.2 (79)
Another GP in a local practice	12.7 (62)
Another nurse/nurse practitioner in the practice	2.7 (13)
Another nurse/nurse practitioner in another local practice	1.2 (6)
Insert/remove implants	
I insert and remove	14.7 (72)
I only insert	0.6 (3)
I only remove	0.6 (3)
Neither	84.1 (411)
Number of implants inserted in the last month (*n* = 72)	
< 1	5.6 (4)
1–5	73.6 (53)
> 5	20.8 (15)
Received implant training	
No	81.8 (400)
Yes	18.2 (89)
Type of implant training received (*n* = 89)	
Family planning course	59.6 (53)
Other (drug rep, Implanon, GP etc.)	29.2 (26)
Not stated	11.2 (10)
Where do women you consult have implant insertions?	
GP in my practice	83.2 (407)
Private gynaecologist	12.3 (60)
Family planning clinic	9.4 (46)
Another GP in a local practice	8.0 (39)
Public hospital	6.3 (31)
Another nurse/nurse practitioner in the practice	5.5 (27)
Another nurse/nurse practitioner in another local practice	1.8 (9)
How often would you initiate discussions about LARC in your contraceptive consultations?	
Never	10.4 (51)
Rarely	25.4 (124)
Sometimes	38.5 (188)
Very often	20.9 (102)
Always	4.9 (24)

Most respondents indicated that patients they consult with had their LARC inserted by a GP in their practice (IUD: 65.2%; implant 83.2%) or a private gynaecologist (IUD: 28.0%; implant 12.3%) (Table 4). Approximately, one quarter of respondents ‘very often’/‘always’ initiated discussion about LARC in their contraceptive consults (25.8%) (Table 4).

## 6 Discussion

Our study was launched at the same time as the AusCAPPS Network, a national online multidisciplinary community of practice that was developed to improve patient access to LARC and medication abortion by supporting Australian primary care clinicians providing these services (Mazza et al. 2022). As part of this work, we sought to describe the LARC knowledge and practices of Australian PNs. Although there is opportunity to better utilise PNs for SRH care, there has been a lack of research indicating their knowledge about and provision of LARC services. Supporting PN provision of these services requires addressing the knowledge gaps identified in this study and enhancing their LARC training, particularly for registered and enrolled PNs. As such, there are important policy and practice implications for the general practice setting to support women's access to LARC services through better PN utilisation.

PNs in our study felt they could influence contraceptive choice for women, yet knowledge was lacking. Similar to international research in general practice about PN training in IUD methods (Hoggart et al. 2018), few in our study had received formal training in LARC. Internationally, SRH knowledge and skills preparation in nursing curricula is limited (Saus‐Ortega et al. 2021; Shi et al. 2025; Uslu Şahan and Yıldırım Hamurcu 2023). This is significant, as we know clinicians are more likely to recommend an efficacious contraceptive method if they have had education and training in its use (Hoggart et al. 2018; Mazza et al. 2017). Although professional interest, organisational and policy support are key issues for SRH in primary care, so is accessing LARC training and funding for the associated costs, especially for rural clinicians (Linton et al. 2023; Mazza et al. 2017). Given that PNs are willing to be better utilised to the extent of practice scope, there needs to be support for them to do so (Australian Primary Health Care Nurses Association 2023). PN training and support in LARC needs to include strategies for service implementation, decision‐making supportive of patient autonomy and managing reproductive coercion by family/partners, and tools to support good clinical practice, such as clinical guidelines and patient resources (Srinivasan et al. 2024). Ongoing PN LARC knowledge can be facilitated by access to evidence‐based SRH resources and further training opportunities, such as through a virtual community of practice (Srinivasan et al. 2024).

Respondent provision of LARC services was low. PNs commonly provide other preventive health initiatives (e.g., immunisation programmes) and report willingness to engage with patients about sexual health topics (Australian Primary Health Care Nurses Association 2023; Leyva‐Moral et al. 2021). PNs also want to utilise existing skills or increase their scope of practice in women's health care (Australian Primary Health Care Nurses Association 2023). Given that most people visit a general practice every year (The Royal Australian College of General Practitioners 2023), the general practice team including PNs, GPs, allied health professionals and reception staff could offer the opportunity for enhanced access to integrated and team‐based women's healthcare. This care should address women's contraceptive needs including increased access to LARC services, advice about pregnancy intervals and the prevention and management of unintended pregnancy (Royal Australian and New Zealand College of Obstetricians and Gynaecologists 2017; World Health Organization 2020).

Similar to other literature, few PNs in our study provided IUDs (Hoggart et al. 2018). Additionally, few PNs in our study provided implants. General practice as a primary place of work meant that respondents were less likely to deliver implant care. Skills maintenance and funding to provide LARC care remain issues for general practice clinicians (Linton et al. 2023). Current funding mechanisms in general practice facilitate GP‐led or supervised care and PN care is geared towards the management of chronic and complex conditions (Australian Government 2024; Australian Government Department of Health and Aged Care 2024; Freund et al. 2015). Yet there are no statutory obligations for GPs to supervise the care nurses provide, and with appropriate funding, alternative models of care can be implemented (AHPRA and National Boards 2022). These models of care need to ensure better utilisation of PNs who are skilled in LARC and encourage those not yet providing LARC to do so, particularly for populations with limited uptake or access, such as adolescents (Bittleston et al. 2024).

### 6.1 Strengths and Limitations

This study examined the LARC knowledge and practices of Australian PNs through a national survey. We had a large representative sample of PHC nurses where demographic information is similar to national workforce data indicating PHC nurses have an average age of 51 years, they largely work in metropolitan areas (59%) and most (79%) are qualified as registered nurses (Australian Primary Health Care Nurses Association 2023). We also used a mix of human and computer‐based verification processes to support the validity of our results.

It is not known how many PNs received the study's invitation and it is likely that only those with an interest in SRH completed the survey. There were a high number of unverified/discontinued respondents excluded from the survey, potentially creating non‐response bias impacting study's results. While our study was based on previous SRH surveys and underwent research team review, we did not use a validated instrument to establish LARC knowledge and practice.

### 6.2 Recommendations for Further Research

Our findings indicate that, although many provide counselling, very few PNs insert LARC in Australia. Future research could examine PN educational pathways, skill development and maintenance opportunities and models of care to enhance the PN provision of LARC care and therefore accessibility of LARC services. In addition, few respondents conducted consultations in a language other than English, indicating further opportunities to research the PN provision of LARC to culturally and linguistically diverse communities.

### 6.3 Implications for Policy and Practice

To improve patient access to LARC services, there is an opportunity to better support PNs to provide this care. Policy solutions for general practice team‐based funding of SRH initiatives are required. In addition, nursing education accreditation can support practice by addressing PHC SRH gaps in nursing curricula. Practice‐based initiatives should include the development of PN‐led LARC models of care.

## 7 Conclusion

PNs experience knowledge gaps and limited practice opportunities to deliver LARC care in general practice. PN access to LARC education and skills‐based training requires development. Although most PN respondents in our survey indicated that their advice could influence women's contraceptive choices, many were unaware that the IUD is a suitable option for nulliparous women, particularly among PNs with less experience and fewer qualifications. This limits the advice PNs can give about the contraceptive options to suit women's reproductive goals. Similarly, the general practice setting limited PN LARC provision, likely due to the focus of PN work on chronic and complex care. This setting and its funding drivers need to better facilitate PN models of care to enhance patient access to LARC.

## Author Contributions

S.J., S.M., S.M.G., K.I.B., D.B., A.T., W.V.N. and D.M.: made substantial contributions to conception and design or acquisition of data or analysis and interpretation of data. S.J., S.M., S.M.G., K.I.B., D.B., A.T., W.V.N. and D.M.: involved in drafting the manuscript or revising it critically for important intellectual content. S.J., S.M., S.M.G., K.I.B., D.B., A.T., W.V.N. and D.M.: given final approval of the version to be published. Each author should have participated sufficiently in the work to take public responsibility for appropriate portions of the content. S.J., D.M.: agreed to be accountable for all aspects of the work in ensuring that questions related to the accuracy or integrity of any part of the work are appropriately investigated and resolved.

## Conflicts of Interest

S.J. is a Board Director of the Australian PHC Nurses Association. D.B. has provided clinical education about contraception for Mayne Pharma, Bayer and Besins but has not received personal remuneration for these services.

## Supporting information


File S1

File S2

## Data Availability

The data are not publicly available due to privacy or ethical restrictions.

## Endnotes

^1^
People who do not identify as women may also require and experience LARC care.

## References

[jan17020-bib-0001] AHPRA & National Boards . 2022. “Supervised Practice Framework.” Accessed October 11, 2024. https://www.ahpra.gov.au/Resources/Supervised‐practice/Supervised‐practice‐framework.aspx.

[jan17020-bib-0002] Australian Government . 2024. “Practice Nurse Items.” Services Australia. Accessed October 11, 2024. https://www.servicesaustralia.gov.au/mbs‐billing‐rules‐for‐practice‐nurse‐items?context=20.

[jan17020-bib-0003] Australian Government Department of Health . 2018a. “National Women's Health Strategy 2020–2030.” C. o. Australia. https://www1.health.gov.au/internet/main/publishing.nsf/Content/national‐womens‐health‐strategy‐2020‐2030.

[jan17020-bib-0004] Australian Government Department of Health . 2018b. “Stronger Rural Health Strategy – Strengthening the Role of the Nursing Workforce.” Australian Government Department of Health. Accessed July 01, 2021. Department of Health Stronger Rural Health Strategy – Strengthening the Role of the Nursing Workforce.

[jan17020-bib-0005] Australian Government Department of Health and Aged Care . 2021. “Modified Monash Model.” Accessed May 31, 2023. https://www.health.gov.au/topics/rural‐health‐workforce/classifications/mmm.

[jan17020-bib-0006] Australian Government Department of Health and Aged Care . 2022. “Health Workforce Data.” Australian Government Department of Health and Aged Care. Accessed November 27, 2023. https://hwd.health.gov.au/datatool/.

[jan17020-bib-0007] Australian Government Department of Health and Aged Care . 2023. “About Nurses and Midwives.” Accessed December 01, 2023. https://www.health.gov.au/topics/nurses‐and‐midwives/about.

[jan17020-bib-0008] Australian Government Department of Health and Aged Care . 2024. “Medicare Benefits Schedule – Item 707.” Australian Government Department of Health and Aged Care. Accessed October 11, 2024. https://www9.health.gov.au/mbs/fullDisplay.cfm?type=item&q=707.

[jan17020-bib-0009] Australian Primary Health Care Nurses Association . 2023. “APNA Workforce Survey 2022.” Accessed March 12, 2024. https://www.apna.asn.au/profession/apna‐workforce‐survey.

[jan17020-bib-0010] Australian Primary Health Care Nurses Association . n.d. “Role of the General Practice Nurse.” Australian Primary Health Care Nurses Association. Accessed May 19, 2023. https://www.apna.asn.au/profession/what‐is‐primary‐health‐care‐nursing/general‐practice‐nursing.

[jan17020-bib-0011] Bittleston, H. , J. S. Hocking , M. Temple‐Smith , L. Sanci , J. L. Goller , and J. Coombe . 2024. “What Sexual and Reproductive Health Issues Do Young People Want to Discuss With a Doctor, and Why Haven't They Done So? Findings From an Online Survey.” Sexual & Reproductive Healthcare 40: 100966. 10.1016/j.srhc.2024.100966. 38522395

[jan17020-bib-0012] Botfield, J. , S. Lacey , K. Fleming , and K. McGeechan . 2019. “Increasing the Accessibility of Long‐Acting Reversible Contraceptives Through Nurse‐Led Insertions: A Cost‐Benefit Analysis.” Collegian 27, no. 1: 109–114. 10.1016/j.colegn.2019.05.001.

[jan17020-bib-0013] Eeckhaut, M. C. , M. M. Sweeney , and J. D. Gipson . 2014. “Who Is Using Long‐Acting Reversible Contraceptive Methods? Findings From Nine Low‐Fertility Countries.” Perspectives on Sexual and Reproductive Health 46, no. 3: 149–155. 10.1363/46e1914. 25040454 PMC4167921

[jan17020-bib-0014] Eysenbach, G. 2004. “Improving the Quality of Web Surveys: The Checklist for Reporting Results of Internet E‐Surveys (CHERRIES).” Journal of Medical Internet Research 6, no. 3: e34. 10.2196/jmir.6.3.e34. 15471760 PMC1550605

[jan17020-bib-0015] Freund, T. , C. Everett , P. Griffiths , C. Hudon , L. Naccarella , and M. Laurant . 2015. “Skill Mix, Roles and Remuneration in the Primary Care Workforce: Who Are the Healthcare Professionals in the Primary Care Teams Across the World?” International Journal of Nursing Studies 52, no. 3: 727–743. 10.1016/j.ijnurstu.2014.11.014. 25577306

[jan17020-bib-0016] Grzeskowiak, L. E. , H. Calabretto , N. Amos , D. Mazza , and J. Ilomaki . 2021. “Changes in Use of Hormonal Long‐Acting Reversible Contraceptive Methods in Australia Between 2006 and 2018: A Population‐Based Study.” Australian & New Zealand Journal of Obstetrics & Gynaecology 61, no. 1: 128–134. 10.1111/ajo.13257. 33095452

[jan17020-bib-0017] Guttmacher Institute . 2019. “Unintended Pregnancy in the United States.” Guttmacher Institute. Accessed February 19, 2025. https://www.guttmacher.org/fact‐sheet/unintended‐pregnancy‐united‐states.

[jan17020-bib-0018] Harris, P. A. , R. Taylor , B. L. Minor , et al. 2019. “The REDCap Consortium: Building an International Community of Software Platform Partners.” Journal of Biomedical Informatics 95: 103208. 10.1016/j.jbi.2019.103208. 31078660 PMC7254481

[jan17020-bib-0019] Harris, P. A. , R. Taylor , R. Thielke , J. Payne , N. Gonzalez , and J. G. Conde . 2009. “Research Electronic Data Capture (REDCap)—A Metadata‐Driven Methodology and Workflow Process for Providing Translational Research Informatics Support.” Journal of Biomedical Informatics 42, no. 2: 377–381. 10.1016/j.jbi.2008.08.010. 18929686 PMC2700030

[jan17020-bib-0020] Health Education England . 2017. The General Practice Nursing Workforce Development Plan. Health Education England. https://www.hee.nhs.uk/our‐work/general‐practice‐nursing.

[jan17020-bib-0021] Hoggart, L. , S. Walker , V. L. Newton , and M. Parker . 2018. “Provider‐Based Barriers to Provision of Intrauterine Contraception in General Practice.” BMJ Sexual & Reproductive Health 44, no. 2: 82–89. 10.1136/bmjsrh-2017-101805. 29921629

[jan17020-bib-0022] Horwood, J. , E. Brangan , P. Manley , et al. 2020. “Management of Chlamydia and Gonorrhoea Infections Diagnosed in Primary Care Using a Centralised Nurse‐Led Telephone‐Based Service: Mixed Methods Evaluation.” BMC Family Practice 21, no. 1: 265. 10.1186/s12875-020-01329-0. 33302884 PMC7731735

[jan17020-bib-0023] James, S. , A. Kunnel , J. Tomnay , D. Mazza , and L. Grzeskowiak . 2023. “Long‐Acting Reversible Contraception Prescribing Coverage by Nurse Practitioners and Midwives in Australia.” Collegian 30, no. 4: 627–632. 10.1016/j.colegn.2023.04.004.

[jan17020-bib-0024] Jones, H. E. , K. O'Connell White , W. V. Norman , E. Guilbert , E. S. Lichtenberg , and M. Paul . 2017. “First Trimester Medication Abortion Practice in the United States and Canada.” PLoS One 12, no. 10: e0186487. 10.1371/journal.pone.0186487. 29023594 PMC5638562

[jan17020-bib-0025] Leyva‐Moral, J. M. , M. Aguayo‐Gonzalez , P. A. Palmieri , G. Guevara‐Vasquez , N. Granel‐Grimenez , and A. Dalfó‐Pibernat . 2021. “Attitudes and Beliefs of Nurses and Physicians About Managing Sexual Health in Primary Care: A Multi‐Site Cross‐Sectional Comparative Study.” Nursing Open 8, no. 1: 404–414. 10.1002/nop2.641. 33318848 PMC7729806

[jan17020-bib-0026] Linton, E. , R. Mawson , V. Hodges , and C. A. Mitchell . 2023. “Understanding Barriers to Using Long‐Acting Reversible Contraceptives (LARCs) in Primary Care: A Qualitative Evidence Synthesis.” BMJ Sexual & Reproductive Health 49, no. 4: 282–292. 10.1136/bmjsrh-2022-201560. 36810206

[jan17020-bib-0027] Mazza, D. , D. Bateson , M. Frearson , P. Goldstone , G. Kovacs , and R. Baber . 2017. “Current Barriers and Potential Strategies to Increase the Use of Long‐Acting Reversible Contraception (LARC) to Reduce the Rate of Unintended Pregnancies in Australia: An Expert Roundtable Discussion.” Australian and New Zealand Journal of Obstetrics and Gynaecology 57, no. 2: 206–212. 10.1111/ajo.12587. 28294293

[jan17020-bib-0028] Mazza, D. , K. Black , A. Taft , et al. 2016. “Increasing the Uptake of Long‐Acting Reversible Contraception in General Practice: The Australian Contraceptive ChOice pRoject (ACCORd) Cluster Randomised Controlled Trial Protocol.” BMJ Open 6, no. 10: e012491. 10.1136/bmjopen-2016-012491. PMC5073472 27855100

[jan17020-bib-0029] Mazza, D. , S. James , K. Black , et al. 2022. “Increasing the Availability of Long‐Acting Reversible Contraception and Medical Abortion in Primary Care: The Australian Contraception and Abortion Primary Care Practitioner Support Network (AusCAPPS) Cohort Study Protocol.” BMJ Open 12, no. 12: e065583. 10.1136/bmjopen-2022-065583. PMC9756212 36521891

[jan17020-bib-0030] Mazza, D. , M. Shankar , J. R. Botfield , et al. 2023. “Improving Rural and Regional Access to Long‐Acting Reversible Contraception and Medical Abortion Through Nurse‐Led Models of Care, Task‐Sharing and Telehealth (ORIENT): A Protocol for a Stepped‐Wedge Pragmatic Cluster‐Randomised Controlled Trial in Australian General Practice.” BMJ Open 13, no. 3: e065137. 10.1136/bmjopen-2022-065137. PMC10040016 36948556

[jan17020-bib-0031] Mid‐Central District Health Board and New Zealand Nurses Organisation Tōpūtanga Tapuhi Kaitaki O Aotearoa and the New Zealand College of Primary Health Care Nurses . 2014. Aotearoa New Zealand Primary Health Care Nursing Standards of Practice. New Zealand Nurses Organisation (Updated 2019). https://www.nzno.org.nz/Portals/0/publications/Primary%20Health%20Care%20Nursing%20Standards%20of%20Practice%202019.pdf?ver=XYUZI2v‐cpVH28Oy1rhfdw%3D%3D.

[jan17020-bib-0032] Mills, J. , J. Chamberlain‐Salaun , L. Christie , M. Kingston , E. Gorman , and C. Harvey . 2012. “Australian Nurses in General Practice, Enabling the Provision of Cervical Screening and Well Women's Health Care Services: A Qualitative Study.” BMC Nursing 11, no. 1: 23. 10.1186/1472-6955-11-23. 23145901 PMC3514301

[jan17020-bib-0033] Norman, W. V. , E. R. Guilbert , C. Okpaleke , et al. 2016. “Abortion Health Services in Canada: Results of a 2012 National Survey.” Canadian Family Physician 62, no. 4: e209–e217. 28192276 PMC4830677

[jan17020-bib-0034] Richters, J. 2016. “Contraceptive Practices Among Women: The Second Australian Study of Health and Relationships.” Contraception 94, no. 5: 548–555. 10.1016/j.contraception.2016.06.016. 27373543

[jan17020-bib-0035] Rowe, H. , S. Holton , M. Kirkman , et al. 2016. “Prevalence and Distribution of Unintended Pregnancy: The Understanding Fertility Management in Australia National Survey.” Australian and New Zealand Journal of Public Health 40, no. 2: 104–109. 10.1111/1753-6405.12461. 26456762

[jan17020-bib-0036] Royal Australian and New Zealand College of Obstetricians and Gynaecologists . 2017. Consensus Statement; Reducing Unintended Pregnancy for Australian Women Through Increased Access to Long‐Acting Reversible Contraceptive Methods. Royal Australian and New Zealand College of Obstetricians and Gynaecologists. Accessed September 22, 2022. https://ranzcog.edu.au/wp‐content/uploads/2022/05/Long‐Acting‐Reversible‐Contraception‐LARC‐Consensus‐Statement.pdf.

[jan17020-bib-0037] Saus‐Ortega, C. , M. L. Ballestar‐Tarín , E. Chover‐Sierra , and A. Martínez‐Sabater . 2021. “Contents of the Sexual and Reproductive Health Subject in the Undergraduate Nursing Curricula of Spanish Universities: A Cross‐Sectional Study.” International Journal of Environmental Research and Public Health 18, no. 21: 11472. 10.3390/ijerph182111472. 34769987 PMC8583184

[jan17020-bib-0038] Shi, Y. , E. Fooladi , J. A. Dean , and S. James . 2025. “Sexual and Reproductive Health Content in Australian Pre‐Registration Nursing and Midwifery Programs: A Review of Curricula.” Nurse Education in Practice 83: 104267. 10.1016/j.nepr.2025.104267. 39864270

[jan17020-bib-0039] Srinivasan, S. , S. M. James , J. Kwek , et al. 2024. “What Do Australian Primary Care Clinicians Need to Provide Long‐Acting Reversible Contraception and Early Medical Abortion? A Content Analysis of a Virtual Community of Practice.” BMJ Sexual & Reproductive Health 51: 94–101. 10.1136/bmjsrh-2024-202330. PMC12013578 38960413

[jan17020-bib-0040] StataCorp . 2023. Stata Statistical Software: Release 18. College Station.

[jan17020-bib-0041] Taft, A. J. , M. Shankar , K. I. Black , D. Mazza , S. Hussainy , and J. C. Lucke . 2018. “Unintended and Unwanted Pregnancy in Australia: A Cross‐Sectional, National Random Telephone Survey of Prevalence and Outcomes.” Medical Journal of Australia 209, no. 9: 407–408. 10.5694/mja17.01094. 30282564

[jan17020-bib-0042] The Faculty of Sexual & Reproductive Healthcare . 2019. UK Medical Eligibility Criteria for Contraceptive Use UKMEC 2016 (Amended September 2019). F. o. S. a. R. Healthcare. https://www.fsrh.org/Common/Uploaded%20files/Standards‐and‐Guidance/fsrh‐ukmec‐full‐book‐2019.pdf.

[jan17020-bib-0043] The Royal Australian College of General Practitioners . 2023. General Practice: Health of the Nation 2023. RACGP. https://www.racgp.org.au/getmedia/122d4119‐a779‐41c0‐bc67‐a8914be52561/Health‐of‐the‐Nation‐2023.pdf.aspx.

[jan17020-bib-0044] The Royal Australian College of General Practitioners . 2024. General Practice: Health of the Nation 2024. RACGP. https://www.racgp.org.au/FSDEDEV/media/documents/Health‐of‐the‐Nation‐2024.pdf.

[jan17020-bib-0045] United Nations Department of Economic and Social Affairs Population Division . 2022. World Family Planning 2022: Meeting the Changing Needs for Family Planning: Contraceptive Use by Age and Method. U. Nations. https://www.un.org/development/desa/pd/sites/www.un.org.development.desa.pd/files/files/documents/2023/Feb/undesa_pd_2022_world‐family‐planning.pdf.

[jan17020-bib-0046] Uslu Şahan, F. , and S. Yıldırım Hamurcu . 2023. “Sexual and Reproductive Health in Nursing Undergraduate Program Curriculums in Turkey: A Cross‐Sectional Study.” Mediterranean Nursing and Midwifery 3, no. 3: 157–164. 10.4274/MNM.2023.23163.

[jan17020-bib-0047] Wood, A. , S. Braat , M. Temple‐Smith , et al. 2021. “A Chlamydia Education and Training Program for General Practice Nurses: Reporting the Effect on Chlamydia Testing Uptake.” Australian Journal of Primary Health 27, no. 1: 36–42. 10.1071/PY20056. 33526167

[jan17020-bib-0048] World Health Organization . 2020. Family Planning/Contraception Methods. World Health Organization. Accessed May 12, 2023. https://www.who.int/news‐room/fact‐sheets/detail/family‐planning‐contraception.

